# The relationship between intolerance of uncertainty and anxiety in autism: A systematic literature review and meta-analysis

**DOI:** 10.1177/1362361320932437

**Published:** 2020-06-22

**Authors:** Richard Jenkinson, Elizabeth Milne, Andrew Thompson

**Affiliations:** 1 The University of Sheffield, UK; 2 Cardiff University, UK

**Keywords:** anxiety, ASD, autism, autism spectrum disorders, intolerance of uncertainty, meta-analysis, systematic review

## Abstract

The association between intolerance of uncertainty and anxiety has proved robust in neurotypical populations and has led to effective interventions targeting intolerance of uncertainty. The aim of this systematic review and meta-analysis was to investigate this association in autistic people, given the high prevalence of anxiety in this population and the limited effectiveness of therapies used currently to treat anxiety in autism. A protocol was published on the Prospero database (CRD42019125315), and electronic databases were searched using terms related to intolerance of uncertainty, anxiety and autism. Included in the systematic review were 12 studies, of which 10 were included in a meta-analysis. Results showed that anxiety and intolerance of uncertainty were consistently elevated in autistic participants. Examining the correlation between these two constructs, the meta-analysis revealed a large sample-weighted effect size, *r* = 0.62, 95% confidence interval = [0.52, 0.71], *p* < 0.001. The strength of this association was comparable to meta-analyses conducted on neurotypical populations, and therefore, it was concluded intolerance of uncertainty may be an appropriate target for intervention for autistic individuals. However, conclusions were limited due to the small number of relevant studies that were available and due to issues with methodological quality.

**Lay abstract:**

People who find it especially hard to cope with the unexpected or unknown are said to have an intolerance of uncertainty. Autistic individuals often report a preference for certainty and experience levels of anxiety that can interfere with their daily life. Understanding more about the link between the intolerance of uncertainty and anxiety in autistic people might lead to better treatments for anxiety being developed. Therefore, this work aimed to review previous research in order to explore this link. Twelve studies were found and their results were compared and contrasted. The autistic people who participated in the studies completed questionnaires that suggested a large number of them experienced very high levels of anxiety and intolerance of uncertainty. Of 10 studies that used relevant statistics, nine found a statistically significant link between anxiety and the intolerance of uncertainty. In general, the strength of the link was about the same as previous research found in people who did not have a diagnosis of autism. This might mean that interventions that aim to help people who are intolerant of uncertainty could be effective for autistic individuals.

## Introduction

Diagnostically, autism spectrum disorder (ASD) is characterised by significant difficulties with social communication/interaction and restricted, repetitive behaviours (American Psychiatric Association, 2013). As it is a neurodevelopmental diagnosis, difficulties are required to have been present during the individual’s early life, even though they may become more pronounced as demands and expectations increase with age.

### Anxiety and ASD

It is estimated that just under half of all autistic people have experienced a comorbid anxiety disorder at some point. For example, separate meta-analyses focusing on prevalence of co-occurring anxiety disorder in children and in adults identified that 40% of children with ASD had at least one comorbid anxiety disorder (van Steensel et al., 2011), and reported a lifetime prevalence of an anxiety disorder in 42% of autistic adults (Hollocks et al., 2019). Specific phobia was more common in children than in adults, whereas obsessive-compulsive disorder and social anxiety disorder were common in both children and adults. Anxiety amplifies difficulties with social functioning in this population and is predictive of poorer quality of life (van Steensel et al., 2012; White et al., 2009). Furthermore, research into the effectiveness of treatments for anxiety has shown high nonresponse rates in autistic individuals (e.g. Storch et al., 2013, 2015; Wood et al., 2015). Therefore, as White et al. (2009) argued, a more thorough understanding of the mechanism(s) underpinning anxiety in this population is required to inform targeted treatments.

### Intolerance of uncertainty

*Intolerance of uncertainty* (IoU) is a trait characterised by the overvaluation of predictability and the tendency to become overwhelmed by the unexpected or the unknown (Birrell et al., 2011; Carleton, 2016; Koerner & Dugas, 2006). In neurotypical populations, IoU is recognised as a dispositional risk factor in the development of generalised anxiety disorder (Carleton et al., 2012; Freeston et al., 1994) and plays a substantial role in social anxiety (Boelen & Reijntjes, 2009; Teale Sapach et al., 2015), obsessive-compulsive disorder (Holaway et al., 2006; Lind & Boschen, 2009; Tolin et al., 2003) and depression (Carleton et al., 2012; de Jong-Meyer et al., 2009; McEvoy & Mahoney, 2012; Nelson et al., 2014). Across diagnostic groups, meta-analytic studies have revealed a robust association between IoU and anxiety in children (Osmanağaoğlu et al., 2018) and in adults (Gentes & Ruscio, 2011). Increased understanding of the association has led to interventions that have aimed to increase tolerance of uncertainty and these have demonstrated effectiveness in the treatment of anxiety (e.g. Dugas et al., 2003; Ladouceur et al., 2000).

### Uncertainty and anxiety in ASD

For many autistic individuals, even slight uncertainty is reported to lead to distress and anxiety, which exacerbates difficulties with social interaction (Ashburner et al., 2013; Bogdashina & Casanova, 2016; Trembath et al., 2012). These qualitative accounts are supported by a limited number of empirical studies. For example, Ivey et al. (2004) found that autistic children showed increased participation in novel social events when they knew what to expect beforehand, and Ferrara and Hill (1980) demonstrated that autistic children were more likely to interact with toys if they could predict when the toys would be revealed to them.

Only recently has the construct of IoU been directly investigated in ASD. To our knowledge, the first study on this topic was published in 2013 by Chamberlain et al. who investigated IoU in a group of 18 autistic adolescents and found that IoU was significantly higher in the autistic adolescents than in a comparison group of neurotypical adolescents (Chamberlain et al., 2013). This work was followed by Boulter et al. (2014) who demonstrated that IoU and anxiety were significantly elevated in the group of autistic children/adolescents in the sample (compared with a neurotypical group). However, once IoU was controlled for, the variance in anxiety accounted for by diagnosis was no longer significant, suggesting IoU might mediate the association between autism and anxiety. In addition, results from additional analyses conducted by the study authors suggested that the relationship between IoU and anxiety functioned similarly in neurotypical and autistic people. This might mean that autistic individuals who experience debilitating anxiety could benefit from interventions targeting IoU.

### The current review

The research into the association between IoU and anxiety in autistic people is still in its infancy. Nevertheless, researchers have begun piloting anxiety interventions that target IoU in this population (e.g. Rodgers et al., 2017). Given the role that IoU has been shown to play in anxiety disorders, and the potential value of interventions that reduce IoU in autism, this therapeutic approach may hold significant promise. However, the origin of anxiety in autistic people may be different than in neurotypical people; therefore, understanding whether there is a similar relationship between IoU and anxiety in ASD, as has been reported in neurotypical samples, is an important goal. The primary aim of this systematic review and meta-analysis is therefore to examine the strength and pattern of the association between IoU and anxiety in autistic children and adults based on research that is available to date. Further to this, certain variables such as age, gender and intelligence quotient (IQ) may moderate the relationship between IoU and anxiety in ASD, meaning that interventions for anxiety that target IoU may be more useful in certain groups of autistic people than others. The secondary aim of the review and meta-analysis will therefore be to explore the variability in the research and the effect of potential moderators such as age, gender and IQ. There is reason to suspect the association might present differently in people of different ages and abilities because, in typical development, the cognitive faculties required to detect and reflect upon uncertainty are likely to mature with age (Osmanağaoğlu et al., 2018), and because previous meta-analyses (e.g. van Steensel et al., 2011) have shown IQ significantly moderates anxiety and rates of anxiety disorders in autistic individuals. Gender will be explored given Boulter et al. (2014) found gender accounted for a significant degree of variance in child-reported anxiety (independently of IoU). Although the meta-analysis will focus on the association between IoU and anxiety, the narrative synthesis will summarise the broader collection of empirical studies investigating IoU and anxiety in autistic people, to shed further light on the association. Because it is common for data concerning autism to be obtained via a proxy (e.g. by parents/caregivers completing questionnaires about their child’s behaviour), we also aimed to investigate whether or not the relationship between anxiety and IoU was moderated by informant type (self-reported data vs proxy-reported data).

As there is little consensus in regard to behavioural or physiological measures that are valid for assessing anxiety in this population (Lydon et al., 2016; Vasa et al., 2016), this review will parallel meta-analyses conducted with neurotypical populations (e.g. Gentes & Ruscio, 2011; Osmanağaoğlu et al., 2018) by including only questionnaire measures. Where studies report both self- and proxy-reported versions (e.g. child- and parent-reported measures), self-reported data will always take precedence. This is because there is often a discrepancy between self-reported and proxy-reported data in relation to autistic individuals (e.g. Vasa et al., 2018) and that individuals are often better-informants of their IoU (Comer et al., 2009). This parallels the approach taken by Osmanağaoğlu et al. (2018).

### Meta-analysis questions

- What is the strength of the association between IoU and anxiety in autistic people?
- How does the association compare to that observed in neurotypical populations?
- Is the relationship moderated by age, gender, IQ or informant type (self-report vs proxy-report)?

## Method

### Search strategy

Prior to formal commencement of the study, a protocol was published on the Prospero database (http://bit.ly/42019125315). Four electronic databases (Scopus, Web of Science, PsycINFO and MEDLINE) were searched from database inception to 1 March 2019. Two electronic research repositories (White Rose Online and ProQuest) were searched to retrieve unpublished studies, in order to reduce publication bias. The Cochrane Library was searched to identify existing reviews on this research topic. Cited references from eligible articles were searched manually.

The titles, abstracts and keywords of databases were searched using terms related to autism, anxiety and IoU. Database-specific search strings are presented in the Supplemental Appendix A. Table 1 gives an overview of the strategy.

**Table 1.** Overarching search strategy.

‘anxiety’	AND	‘autism’	AND	‘Intolerance’	AND	‘uncertainty’
‘fear’		‘ASD’				
‘GAD’		‘ASC’				
‘OCD’		‘PDD’				
‘compulsive disorder’		‘Asperg*’				
‘panic’		‘pervasive developmental disorder’				
		‘Pathological Demand’				
		‘PDA’				

OR used as operator between items in each column.

### Inclusion/exclusion criteria

To be included, articles were required to have been available in English and to have included original research in which questionnaire measures of both IoU and anxiety were used to report on autistic individuals (either via self-report or via proxy). To be included in the meta-analysis, studies were required to have reported the correlation between IoU and anxiety. However, if these data were not available, studies were still included in the narrative synthesis if they made comparisons between an autistic group and a neurotypical group (on IoU and anxiety). Studies were excluded if they used data from an earlier published study, or if they used single-case designs. The first author screened the articles and abstracts of all articles and reviewed the full text of the remaining articles against the inclusion/exclusion criteria.

### Data extraction

A bespoke form was designed specifically to extract data from studies included in this review. The first author extracted the following data from each study: authors, year of publication, country in which data were collected, study design, recruitment strategy, sample size, research team, category of autism diagnosis, method used to establish/confirm diagnosis, sample characteristics (gender, cognitive measures, age), type of IoU and anxiety measures used, baseline IoU and anxiety scores and the reported correlation between IoU and anxiety. Where authors used a neurotypical comparison group, data were extracted per group. Specific data on socioeconomic status and race/ethnicity were not recorded as a scoping review indicated they were seldom reported in articles meeting the review’s inclusion criteria. There were two studies included in the meta-analysis that met inclusion criteria on the basis of additional data requested directly from authors (supplied via email).

### Quality appraisal

Studies were appraised at the outcome level using a quality appraisal checklist for correlational studies (The National Institute for Health and Clinical Excellence, 2012). Using the checklist, studies were scored on the basis of how well they met criteria for each applicable item (criteria are available in Supplemental Appendix B). Two points were assigned for a study in which criteria were fully met on an item and one point was assigned for a study in which criteria were partially met (zero points were awarded for studies failing to meet an item’s criteria). To aid inter-study comparisons, an overall quality score was calculated for each study by summing item-level scores, dividing by the maximum score achievable, and multiplying by 100. The checklist also included two summary items in which an overall rating of the study’s internal and external validity was made. A double cross (++) was assigned for a summary item if the study fully met criteria, a single cross (+) was used if criteria were partially met, and a minus sign (−) was applicable if criteria were not met. The ratings from this checklist were integrated into the narrative of the review.

The wording of one checklist item (2.3) was changed as it pertained to potential contamination between an exposure and comparison group (which was not relevant to the present review). To fulfil a similar criterion, the revised item specified whether a diagnosis of autism was confirmed independently by the researchers, as this minimised bias by ensuring the study only included autistic participants. Modifying checklists in the manner described above is consistent with guidance from the Centre for Reviews and Dissemination (2008). Please see Supplemental Appendix B for the completed checklist.

### Statistical analyses

Pearson’s product-moment correlation coefficient (*r*) was selected as the effect size for the meta-analysis, due it being easily interpretable and a popular choice for meta analyses between IoU and anxiety conducted with neurotypical populations (e.g. Gentes & Ruscio, 2011; Osmanağaoğlu et al., 2018), facilitating comparisons. Analyses were performed using the software package, Comprehensive Meta-Analysis (Borenstein et al., 2005). A random-effects model was selected due to anticipated heterogeneity between studies and because it permits results to more readily be generalised (Chen & Peace, 2013). To interpret the correlations, guidelines by Cohen (1988) were used to define small, moderate and large effects (*r* = 0.10, *r* = 0.30, *r* = 0.50, respectively), and 95% confidence intervals were calculated. To correct for skewed sampling distribution when population values of *r* move further from zero, correlations were transformed to Fisher’s Z for meta-analytic computations (Cooper & Hedges, 1993).

To aid visual inspection of the data, funnel plots and forest plots were produced. A regression test (Egger et al., 1997) was also used to assess publication bias. Fail-safe analysis (Rosenthal, 1979) was conducted to aid this assessment by quantifying the number of studies that would be required to invalidate the effect (Borenstein et al., 2011).

In order to assess heterogeneity, the *Q* and *I*^2^ statistics were used. Significant results indicate heterogeneity. Higgins et al. (2003) suggested that *I*^2^ percentages of 25%, 50% and 75% can be interpreted as representing low, moderate and high heterogeneity, respectively.

Heterogeneity was explored using potential moderators specified a priori. Meta-regression was planned for numerical moderators (age, percentage male, IQ). Subgroup analyses were planned to examine the effect of informant type and instrument selection on the relationship between IoU and anxiety. However, the latter analysis was not conducted given it was specified a priori that there needed to be at least four studies in each subgroup (Bakermans-Kranenburg et al., 2003).

## Results

**Figure 1.**
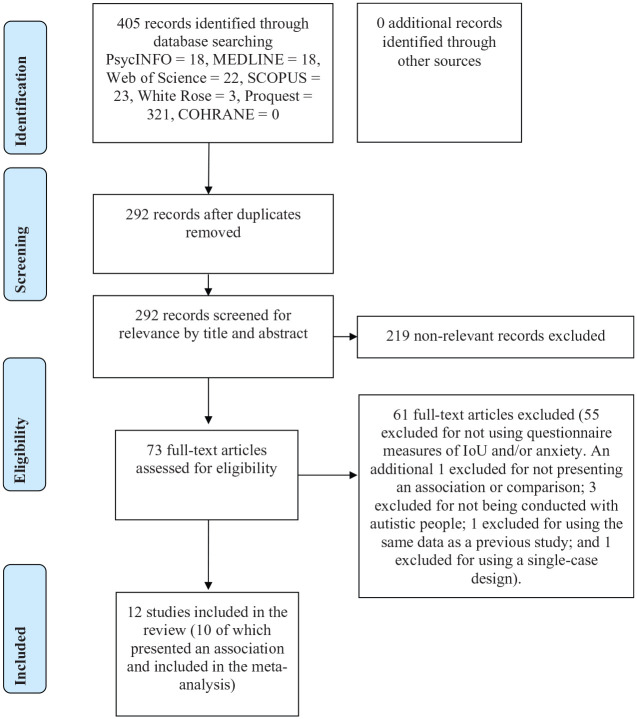
PRISMA flow diagram representing the selection of studies included in the review.

The search retrieved 405 articles. There were 113 duplicates removed, and the remaining 292 articles were screened for relevance. After 219 irrelevant records were excluded, the full text of the remaining 73 articles was reviewed and examined using a priori inclusion and exclusion criteria. There were 12 studies that remained and were included in the literature review (10 of which were included in the meta-analysis). No additional records were identified through manual searches of the reference lists of the 12 included studies. A diagrammatic representation of the process can be seen in Figure 1.

### Participants

The 12 studies included in the review comprised 656 participants (562 in the meta-analysis). Upon scrutinising the research reports (and contacting the researchers for additional clarity where required), there was a high degree of confidence that all studies used independent participants.

The ages of participants were variable, ranging from 4 to 70 years (4–24 years in the meta-analysis). The majority of the studies comprised child and adolescent participants (with ages that ranged from 4 to 18 years). All samples had a high proportion of males (ranging from 70.5% to 94.4%). Dates of publication were all within the last 7 years.

All researchers recruited a convenience sample from Western, English-speaking countries, with the majority opting to recruit participants from a research database. Owing to the origins of the IoU research in people with ASD, there were seven studies which included at least a partial collaboration with the research team at Newcastle University in the United Kingdom.

Eight studies measured full-scale IQ. Participants had a combined mean IQ of 105.4 (*SD* = 15.2). An additional study had nonverbal and verbal IQ scores within one standard deviation of the general population mean, and the remaining three reported excluding participants with intellectual disability. The six studies measuring IQ in the meta-analysis had a combined mean IQ of 103.5 (*SD* = 15.3).

### Instruments and data analysis

There were 10 studies that used a variant of the Intolerance of Uncertainty Scale (Buhr & Dugas, 2002), with the majority of researchers opting to use the 12-item shortened version. The remaining two studies used the IoU subscale from the Anxiety Scale for Children-ASD (ASC-ASD; Rodgers et al., 2016). They met inclusion criteria because both studies included a separate anxiety measure.

To measure anxiety, half of the studies used the Spence Children’s Anxiety Scale (SCAS; Spence, 1998). The second most popular tool was the Screen for Child Anxiety Related Emotional Disorders (SCARED; Birmaher et al., 1997). Additional measures included the Dimensional Anxiety Scales (DAS; American Psychiatric Association, 2013) and the State-Trait Anxiety Inventory (STAI; Spielberger, 2010). All studies used trait measures of IoU and anxiety, and all data extracted from the studies were cross-sectional.

### Quality assessment summary

Overall quality ratings for included studies were variable, with scores ranging from 30% to 82% (38%–82% in the meta-analysis). Given the limited number of studies available, none were excluded on the basis of quality.

Of 12 studies, 11 confirmed participants had a valid diagnosis of ASD, either by using independent gold-standard diagnostic instruments or by confirming self-reports utilising established autism-screening tools and excluding participants who did not meet clinical thresholds. One study (Joyce et al., 2017) used a screening tool but did not exclude participants with subthreshold scores.

None of the studies used an IoU measure that had been validated for autistic people (several authors commented that one was not available). There were seven studies that checked the internal consistency of the IoU measure (values ranged from acceptable to excellent). All seven were included in the meta-analysis. The remaining five did not check. Only one study (Boulter et al., 2014) had missing data that was not accounted for. There were only two studies (Boulter et al., 2014; Rodgers et al., 2016) that reported making an a priori power calculation and achieving adequate power.

A common external validity issue was a lack of detail about the source population, the detail and representativeness of the eligible population, and how the clinical and demographic characteristics of these populations compared with the participants in the sample. A notable exception was Rodgers et al. (2016). There was generally a lack of detail about recruitment and the details of those who were eligible but declined.

### The relationship between IoU and anxiety in autistic people

Keefer et al. (2017) used combined self-reported and parent-reported data to group autistic children into a high and low IoU group. All participants in the high IoU group were found to have clinically significant anxiety at baseline, compared with 65% in the low IoU group.

#### Exclusively self-reported data

Five studies (Boulter et al., 2014; Cai et al., 2018; Joyce et al., 2017; Rodgers et al., 2016; Vasa et al., 2018) reported finding a large, significant association between self-reported anxiety and self-reported IoU. Keefer et al. (2017) reported finding a moderate, significant association.

#### Inclusive of parent-reported data

All four of the studies in which the correlation was based on at least one measure that was parent reported (Damiano, 2015; Glod, 2017; Neil et al., 2016; Wigham et al., 2015) found a significant association between IoU and anxiety of a large effect size.

### ASD as a predictor

Vasa et al. (2018) found a diagnosis of ASD was predictive of IoU and that this was not fully accounted for by the effect of anxiety. Neil et al. (2016) found a large, significant, indirect effect of a diagnosis of ASD on anxiety, through IoU (without an accompanying direct effect). Maisel et al. (2016) used structural equation modelling to investigate this relationship, with scores from the autism spectrum quotient (Baron-Cohen et al., 2001) representing autism severity. Similar to Vasa et al. and Neil et al., the researchers found severity predicted IoU and that IoU partially mediated the association between severity and anxiety (accounting for 36% of the effect). When IoU was controlled for, severity did not predict anxiety.

Two studies (Glod, 2017; Wigham et al., 2015) found sensory hyper-responsiveness correlated significantly with IoU. Furthermore, Wigham et al. (2015) conducted a regression analysis that revealed a significant, serial, indirect effect from sensory-responsiveness through IoU and anxiety to insistence on sameness.

The primary findings of the 12 studies are summarised in Table 2. Pertinent clinical/demographic characteristics are included to aid inter-study comparisons.

**Table 2.** Summary of findings from primary outcome measures.

Study	Country	ASD sample size, gender, IQ (*SD*)	Age (*SD*), range	IoU measure	Anxiety measure	Correlation	Between-group differences in IoU and anxiety	Quality
Boulter et al. (2014)	UK, USA	*N* = 114 88% male IQ = 108.5 (13.8)	12.7 (2.9) 8–18	**IUS-12 (child-SR);** IUS-12 (parent)	**SCAS (child-SR);** SCAS (parent)	0.70** (data from author via email)	Autistic group had significantly higher anxiety than NT group (60 % had clinically significant anxiety, vs 12% of the NT group). Autistic group had significantly higher IoU than NT group.	62% (+,+)
Cai et al. (2018)	Australia	*N* = 61 70% male IQ = NR	18.2 (2.2) 14–24	**IUS-12** **(SR)**	**DSM-5 DAS** (SR)	0.63**		46% (+,+)
Chamberlain et al. (2013)	USA	*N* = 18 94% male IQ = 104.8 (13.3)	16.6 (1.0) 15–18	IUS-12 (Child-SR); IUS-12 (parent)	SCAS (child-SR); SCAS (parent)	Not available so excluded from meta-analysis	Based on parental report, autistic group had significantly higher anxiety and IoU than NT group. However, no significant differences found on child data.	30% (NA, −)
Damiano (2015)	USA	*N* = 26 92% male IQ = 105.69 (17.5)	14.1 (3.2) 9–18	**IUS-27 (child-SR);** IUS-27 (parent)	**SCARED (parent)**	0.16	Autistic group had significantly higher anxiety and IoU than NT group.	38% (−,+)
Glod (2017)	UK	*N* = 19 (parents) 84% male Verbal IQ = 89.7 (13.6)	7.21 (1.8) 4–9	**IoU subscale of ASC-ASD (parent)**	**T-scores from the SCAS/PAS (parent)**	0.83*		42% (+,−)
Joyce et al. (2017)	UK	*N* = 13 84% male IQ = NR	16.8 (2.4) 13–20	**IUS-12 (Child-SR);** IUS-12 (parent)	**SCAS** **(SR)**	0.82**		46% (−,−)
Keefer et al. (2017)	USA	*N* = 43 81% male IQ = 102.6 (14.7)	11.2 (2.0) 8–14	**IUS-27 (child-SR, modified language);** IUS-27 (parent)	**SCARED (child-SR);** SCARED (parent)	0.36*		38% (+,+)
Maisel et al. (2016)	UK, USA	*N* = 76 78% male IQ = 111.2 (15.0)	33.8 (14.9) 17–70	IUS-12 (SR)	STAI-T Form Y (SR)	Not available so excluded from meta-analysis	Based on self-report, autistic group had significantly higher anxiety and IoU than NT group.	55% (NA,−)
Neil et al. (2016)	UK	*N* = 64 (parents) 86% male IQ = 98.6 (14.9)	10.4 (2.4) 6–14	**IUS-12 (parent)**	**SCAS (parent)**	0.74**	Autistic group had significantly higher anxiety and IoU than NT group.	73% (++,+)
Rodgers et al. (2016)	UK	*N* = 112 77% male IQ = NR	11.1 (2.1) 8–15	**IoU subscale of ASC-ASD (child-SR)** IoU subscale of ASC-ASD (parent)	**SCARED (child-SR);** SCARED (parent)	0.72* (data from author via email)		82% (NA,++)
Vasa et al. (2018)	USA	*N* = 57 83% male IQ = 100.9 (14.9)	10.9 (2.0) 7–16	**IUS-27 (child-SR, modified language)** IUS-27 (parent)	**SCARED (child-SR);** SCARED (parent)	0.46*	No significance between self-reported anxiety in autistic and NT groups. Autistic group had significantly higher IoU than NT group.	69% (+,+)
Wigham et al. (2015)	USA, UK	*N* = 53 89% male IQ = 106.2 (14.8)	12.50 (2.3) 8–16	**IUS-12 (parent)**	**SCAS (parent)**	0.57*		58% (+,+)

NR, not reported; ASD, autism spectrum disorder; SR, self-reported; Child-SR, child self-reported version of the measure; Parent, parent-informant version of the measure; NT, neurotypical; IoU, intolerance of uncertainty; IUS-12, Intolerance of Uncertainty Scale (12-item version); IUS-27, Intolerance of Uncertainty Scale (27-item version); DSM-5 DAS, *Diagnostic and Statistical Manual of Mental Disorders* (5th ed.) Dimensional Anxiety Scales; SCAS, Spence Children’s Anxiety Scale; PAS, Preschool Anxiety Scale; ASC-ASD, Anxiety Scale for Children-ASD; SCARED, Screen for Child Anxiety Related Disorders; STAI-T (Form Y), State-Trait Anxiety Inventory (trait version).

Bold typeface denotes the version of the measure used in the correlation calculation.

* significant at *p* < 0.05 level; **significance at *p* < 0.01 level.

### Meta-analysis

**Figure 2.**
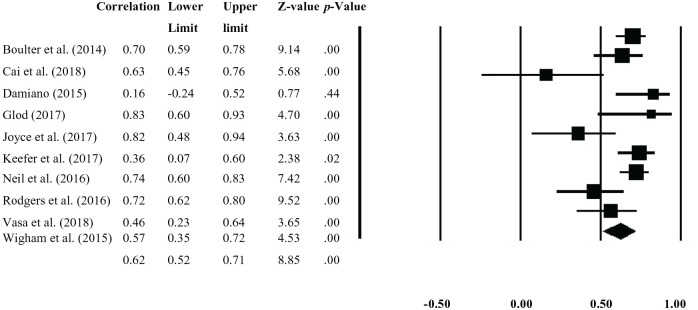
Meta-analysis forest plot.

The sample-weighted effect size was *r* = 0.62, 95% confidence interval = [0.52, 0.71], and significant (*p* < 0.001), which suggested a large, positive correlation between IoU and anxiety (with IoU explaining 38.44% of the variance in anxiety). The *Q* statistic was significant, *Q*(9) = 28.84, *p* = 0.001, and the *I*^2^ (69%) statistic was moderate-high, suggesting heterogeneity in the data. The corresponding Forest Plot is shown in Figure 2.

A funnel plot was produced, with the effect size from each study on the x-axis, and a measure of study precision – indicated by standard error of effect size on the y-axis (see Supplemental Figure 1). The asymmetry of the funnel plot indicated evidence of potential publication bias, and two studies fell outside the 95% confidence limits. However, the low number of studies included in this meta-analysis limited interpretation of this plot. Examining the symmetry statistically via a regression test indicated there was not significant evidence of publication bias (*t*(8) = 0.62, *p* = 0.56). Furthermore, the fail-safe analysis indicated that 679 missing studies would be required to bring the *p* value to >0.05.

Heterogeneity was explored using subgroup analyses and meta-regression. For numerical variables, meta-regression analysis revealed there was not a significant effect of age (*Q*(1) = 0.27, *p* = 0.61) or gender (*Q*(1) = 0.41, *p* = 0.52). Both of these analyses included all studies. For the six studies that provided a full-scale IQ score, meta-regression analysis indicated a significant effect (*Q*(1) = 6.91, *p* = 0.001), such that the relationship between IoU and anxiety strengthened mildly as IQ increased.

The effect of informant on the association between IoU and anxiety was explored using subgroup analysis. Data from studies that were exclusively self-reported (*n* = 6) yielded a pooled-effect size (*r* *=* 0.62, *p* < 0.001) that was virtually identical to the pooled effect of studies (*n* = 4) where the data on at least one measure were parent-reported (*r* *=* 0.63, *p* < 0.001).

## Discussion

This was the first time the research on the association between anxiety and IoU in autistic people was synthesised and analysed in a systematic review and meta-analysis. Of the 10 studies included in the meta-analysis, a significant correlation was found in nine (seven finding a large effect; two finding a moderate effect). All effects were in the same direction, indicating a positive association between IoU and anxiety, that is, higher anxiety was generally found in participants who were more intolerant of uncertainty (and vice versa). There was only one study that did not find a significant correlation, and this was an unpublished dissertation and among the weakest in terms of quality, suggesting caution was advisable when interpreting the result.

The meta-analysis showed a mean effect size that was large and suggested that IoU was associated with 38% of the variance in anxiety among the participants (with ages ranging from 4 to 24 years). This result was consistent with a recent meta-analysis (Osmanağaoğlu et al., 2018) conducted in a neurotypical, Western population with ages ranging from 3 to 20 years, and in which the majority of studies utilised identical or very similar outcome measures to the present review. The researchers found IoU explained 36% of the variance in anxiety. Therefore, the primary conclusion from this review is that the strength of the association between IoU and anxiety in autistic children/young adults appears comparable to that found in neurotypical populations. The effect size of the relationship between IoU and anxiety found here confirms that IoU may be a useful focus for therapeutic interventions targeting anxiety in ASD, as has been suggested for community samples (Osmanağaoğlu et al., 2018). While this meta-analysis cannot speak to the mechanisms that underlie the association between IoU and anxiety in ASD, understanding whether there may be unique mechanisms that link IoU and anxiety in ASD is an important future step. For example, a number of questions remain to be answered, including to what extent do restricted interests and insistence on sameness influence the relationship between IoU and anxiety in ASD, to what extent are restricted interests and insistence on sameness influenced by IoU and anxiety in ASD, and are the mechanisms that link IoU and anxiety different in autistic and neurotypical samples. Significant heterogeneity was found in the present meta-analysis, and potential moderating variables were explored. Using meta-regression analyses, age and gender did not appear to have a significant impact on the effect. The same result was found in the meta-analysis by Osmanağaoğlu et al. (2018). However, as the researchers argued, there are theoretical reasons why it might be expected that the relationship changes as cognitive abilities develop. Although the nonsignificant effect of age went against this prediction, this review differed from Osmanağaoğlu’s as IQ score was also explored here as a potential moderator. A meta-regression analysis found a significant result that suggested the association between IoU and anxiety strengthened mildly as IQ increased. This finding is relevant in the context of a recent meta-analysis by van Steensel and Heeman (2017), as this study found that anxiety levels were elevated in autistic children (compared with neurotypical children) and that this difference widened as IQ increased. The authors suggested it was plausible this was because the autistic children who had increased cognitive functioning had more insight into their difficulties and the demands upon them, leading to anxiety.

The present review included studies (e.g. Maisel et al., 2016) that suggested IoU partially mediated the association between the core features of autism and anxiety, and one study (Wigham et al., 2015) found a significant, serial, indirect effect from sensory-responsiveness through IoU and anxiety to insistence on sameness. This is in line with theories that suggest sameness behaviours may function to reduce short-term anxiety by avoidance of uncertain situations that provoke distress (Joosten et al., 2009). Therefore, perhaps high-functioning autistic individuals have greater insight into their difficulties and this motivates the need for predictability and raises anxiety about the potential impact their difficulties can have on meeting uncertain demands. To reduce this anxiety, individuals may insist on sameness (resulting in a vicious cycle).

Subgroup analysis of the association between IoU and anxiety revealed that studies that used exclusively self-reported data did not differ significantly from studies that included parental-reported data. This was surprising given many studies in this review reported finding inconsistency between parent and child reports. One possible explanation for this is that the degree of inconsistency was consistent across measures of IoU and anxiety. Therefore, although parents might have scored individual measures of IoU and anxiety differently to their children, this did not have a significant impact on the *relationship* between them.

### Limitations

This review’s reliance on questionnaire measures of IoU and anxiety was a limitation, especially given the lack of previous research that has validated measures of IoU in samples of autistic people. Further research is necessary to ascertain whether the measures are valid and reliable, before the results from studies using them can be interpreted with confidence. Furthermore, the quality of the studies was compromised on a number of variables (the representativeness of the participants, the precision of the effect sizes reported, etc.). It is therefore a limitation that all studies were included in the meta-analysis, irrespective of their quality. The decision to include all studies was based on the relatively small number of studies that have investigated the relationship between IoU and anxiety. Given the consistency of findings across all of the studies included in the meta-analysis, we do not think that excluding the poorer quality studies from the meta-analysis would change the overall findings substantially. Nevertheless, it is hoped that any future work in this area is able to overcome some of the limitations of currently published work.

Although this review aimed to synthesise data across the life span, only one study used adult participants exclusively, and this was excluded from the meta-analysis. Subsequently, it was not possible to make inferences about how the relationship between IoU and anxiety develops into adulthood. The findings from this analysis are only relevant to children and young adults therefore. There was also a high percentage of males in the included studies, although this proportion was consistent with prevalence estimates (Whiteley et al., 2010). Finally, the small number of studies included in the review and the cross-sectional nature of the studies limited the conclusions that could be drawn.

## Conclusion

Boulter et al. (2014) found evidence that suggested IoU may mediate the relationship between autism and anxiety and that the relationship between IoU and anxiety is similar in neurotypical and autistic individuals. In their discussion, the researchers recommended that anxiety interventions be developed that target IoU in autistic people. The current review adds to the growing evidence base in support of this proposal by demonstrating that IoU and anxiety are consistently elevated in autistic people and that the strength of the relationship is comparable to neurotypical populations. This might mean IoU is an appropriate target for intervention in autistic individuals, as it is in neurotypical populations.

It is encouraging that IoU interventions are currently being developed for autistic people and that preliminary data are promising (e.g. Rodgers et al., 2017). However, this review has highlighted steps that would potentially strengthen the quality of work in this field (e.g. the validation of IoU measures).

## Supplemental Material

Supplemental_material_revised_2 – Supplemental material for The relationship between intolerance of uncertainty and anxiety in autism: A systematic literature review and meta-analysis Click here for additional data file. Supplemental material, Supplemental_material_revised_2 for The relationship between intolerance of uncertainty and anxiety in autism: A systematic literature review and meta-analysis by Richard Jenkinson, Elizabeth Milne and Andrew Thompson in Autism
