# Supplementary material for: The relationship between intolerance of uncertainty and anxiety in autism: A systematic literature review and meta-analysis
Source: Autism. 2020 Jun 22;24(8):1933–44. doi: 10.1177/1362361320932437 (PMC7539603; doi:10.1177/1362361320932437)

Supplementary Material

Appendix A

Search Strategy

PsycINFO

(("anxiety" or "fear" or "GAD" or "OCD" or "compulsive disorder" or "panic") and ("autism" or "ASD" or "ASC" or "PDD" or "Asperg*" or "pervasive developmental disorder" or "Pathological Demand" or "PDA") and "intolerance" and "uncertainty").ab. or (("anxiety" or "fear" or "GAD" or "OCD" or "compulsive disorder" or "panic") and ("autism" or "ASD" or "ASC" or "PDD" or "Asperg*" or "pervasive developmental disorder" or "Pathological Demand" or "PDA") and "intolerance" and "uncertainty").ti. or (("anxiety" or "fear" or "GAD" or "OCD" or "compulsive disorder" or "panic") and ("autism" or "ASD" or "ASC" or "PDD" or "Asperg*" or "pervasive developmental disorder" or "Pathological Demand" or "PDA") and "intolerance" and "uncertainty").id.

MEDLINE (include related terms)

(("anxiety" or "fear" or "GAD" or "OCD" or "compulsive disorder" or "panic") and ("autism" or "ASD" or "ASC" or "PDD" or "Asperg*" or "pervasive developmental disorder" or "Pathological Demand" or "PDA") and "intolerance" and "uncertainty").ab. or (("anxiety" or "fear" or "GAD" or "OCD" or "compulsive disorder" or "panic") and ("autism" or "ASD" or "ASC" or "PDD" or "Asperg*" or "pervasive developmental disorder" or "Pathological Demand" or "PDA") and "intolerance" and "uncertainty").ti. or (("anxiety" or "fear" or "GAD" or "OCD" or "compulsive disorder" or "panic") and ("autism" or "ASD" or "ASC" or "PDD" or "Asperg*" or "pervasive developmental disorder" or "Pathological Demand" or "PDA") and "intolerance" and "uncertainty").id.

SCOPUS (title, abstract, keywords)

("anxiety" OR "fear" OR "GAD" OR "OCD" OR "compulsive disorder" OR "panic") AND (“intolerance of uncertainty”) AND ("autism" OR "ASD" OR "ASC" OR "PDD" OR "Asperg*" OR "pervasive developmental disorder" OR "Pathological Demand" OR "PDA")

WEB OF SCIENCE

TOPIC: ("anxiety" or "fear" or "GAD" or "OCD" or "compulsive disorder" or "panic") AND TOPIC: ("autism" or "ASD" or "ASC" or "PDD" or "Asperg*" or "pervasive developmental disorder" or "Pathological Demand" or "PDA") AND TOPIC: ("intolerance of uncertainty")

Timespan: All years. Databases: WOS, BCI, BIOSIS, CCC, DRCI, DIIDW, KJD, MEDLINE, RSCI, SCIELO, ZOOREC. Search language=Auto

White Rose

Title (any of): autism, ASD, ASC, PDD, Asperg*, pervasive developmental disorder, Pathological Demand, PDA

+ Abstract (all of):intolerance of uncertainty

PROQUEST

IN anywhere:

("anxiety" OR "fear" OR "GAD" OR "OCD" OR "compulsive disorder" OR "panic") AND (“intolerance of uncertainty”) AND ("autism" OR "ASD" OR "ASC" OR "PDD" OR "Asperg*" OR "pervasive developmental disorder" OR "Pathological Demand" OR "PDA")

Appendix B

Quality Table

	Boulter, Freeston, South, & Rodgers (2014)	Cai, Richdale, Dissanayake, & Uljarević (2018)	Chamberlain et al. (2013)	Damiano (2015)	Glod (2017)	Joyce, Honey, Leekam, Barrett, & Rodgers (2017)	Keefer et al. (2017)	Maisel et al. (2016)	Neil, Olsson, & Pellicano (2016)	Rodgers et al. (2016)	Vasa, Kreiser, Keefer, Singh, & Mostofsky (2018)	Wigham, Rodgers, South, McConachie, & Freeston (2015)
**1.1 Is the source population or source area well described?** Was the country (e.g. developed or non-developed, type of health care system), setting (primary schools, community centres etc), location (urban, rural), population demographics etc adequately described?	+	+	-	+	+	+	+	+	+	++	+	+
**1.2 Is the eligible population or area representative of the source population or area?** Was the recruitment of individuals, clusters or areas well defined (e.g. advertisement, birth register)? Was the eligible population representative of the source?	+	+	-	-	+	+	+	+	+	++	+	+

Were important groups underrepresented**?**												
**1.3 Do the selected participants or areas represent the eligible population or area?** Was the method of selection of participants from the eligible population well described? What % of selected individuals or clusters agreed to participate? Were there any sources of bias? Were the inclusion or exclusion criteria explicit and appropriate?	-	-	-	-	-	-	-	-	-	++	+	-
2.1 **Selection of exposure (and comparison) group. How was selection bias minimised?**	NA	NA	NA	NA	NA	NA	NA	NA	NA	NA	NA	NA
**2.2 Was the selection of explanatory variables based on a sound theoretical basis?**	++	++	++	++	++	++	++	++	++	++	++	++
**2.3 Was the diagnosis of autism confirmed by the researchers ?** Did they use a gold-standard diagnostic measure?	+	+	++	++	+	-	+	++	++	+	++	+
**2.4 How well were likely confounding factors identified and controlled?** Were there likely to be other confounding factors not considered or appropriately adjusted for? Was this sufficient to cause important bias?	+	+	NA	+	-	-	-	NA	+	NA	++	+

**2.5 Is the setting applicable to the UK?** Did the setting differ significantly from the UK?	+	+	+	+	++	++	+	+	++	++	+	+
**3.1 Were the outcome measures and procedures reliable?** Were outcome measures subjective or objective (e.g. biochemically validated nicotine levels ++ vs self-reported smoking −)? How reliable were outcome measures (e.g. inter- or intra-rater reliability scores)? Was there any indication that measures had been validated (e.g. validated against a gold standard measure or assessed for content validity)?	+	+	-	-	-	+	-	-	+	+	-	-
**3.2 Were the outcome measurements complete?** Were all or most of the study participants who met the defined study outcome definitions likely to have been identified?	-	+	+	+	+	++	++	+	++	+	++	++
**3.3 Were all the important outcomes assessed?** Were all the important benefits and harms assessed? Was it possible to determine the overall balance of benefits and harms of the intervention versus comparison?	NA	NA	NA	NA	NA	NA	NA	NA	NA	NA	NA	NA

**3.4 Was there a similar follow-up time in exposure and comparison groups?** If groups are followed for different lengths of time, then more events are likely to occur in the group followed-up for longer distorting the comparison. Analyses can be adjusted to allow for differences in length of follow-up (e.g. using person-years).	NA	NA	NA	NA	NA	NA	NA	NA	NA	NA	NA	NA
**3.5 Was follow-up time meaningful?** Was follow-up long enough to assess long-term benefits and harms? Was it too long, e.g. participants lost to follow-up?	NA	NA	NA	NA	NA	NA	NA	NA	NA	NA	NA	NA
**4.1 Was the study sufficiently powered to detect an effect (if one exists)?** A power of 0.8 (i.e. it is likely to see an effect of a given size if one exists, 80% of the time) is the conventionally accepted standard. Is a power calculation presented? If not, what is the expected effect size? Is the sample size adequate?	++	+	-	-	+	+	+	+	+	++	+	+

**4.2 Were multiple explanatory variables considered in the analyses?**	++	+	NA	++	-	+	-	++	++	NA	++	++
**4.3 Were the analytical methods appropriate?** Were important differences in follow-up time and likely confounders adjusted for?	++	+	NA	-	+	+	+	NA	++	+	++	++
**4.6 Was the precision of association given or calculable? Is association meaningful?** Were confidence intervals or p values for effect estimates given or possible to calculate? Were CIs wide or were they sufficiently precise to aid decision-making? If precision is lacking, is this because the study is under-powered?	++	+	-	-	+	-	-	+	++	++	+	+
**OVERALL QUALITY SCORE**	62%	46%	30%	38%	42%	46%	38%	55%	73%	82%	69%	58%
**5.1 Are the study results internally valid (i.e. unbiased)?** How well did the study minimise sources of bias (i.e. adjusting for potential confounders)? Were there significant flaws in the study design?	+	+	NA	-	+	-	+	NA	++	NA	+	+
**5.2 Are the findings generalisable to the source population (i.e. externally valid)?** Are there sufficient details given about the study to determine if the findings are generalisable to the source	+	+	-	+	_	_	+	-	+	++	+	+

population? Consider: participants, interventions and comparisons, outcomes, resource and policy implications.												

Note: Criteria fully met (++) , criteria partially met (+), criteria not met (-), not applicable (NA) and item not counted in percentage for particular study. Overall quality score is calculated as a percentage by dividing the study's points by the total possible for the particular study and multiplying by 100. Summary items (5.1. and 5.2) are not included in the percentage.

Appendix C

PRISMA Checklist

Section/topic	#	Checklist item	Reported on page #
**TITLE**			
Title	1	Identify the report as a systematic review, meta-analysis, or both.	1
**ABSTRACT**			
Structured summary	2	Provide a structured summary including, as applicable: background; objectives; data sources; study eligibility criteria, participants, and interventions; study appraisal and synthesis methods; results; limitations; conclusions and implications of key findings; systematic review registration number.	2
**INTRODUCTION**			
Rationale	3	Describe the rationale for the review in the context of what is already known.	3-5
Objectives	4	Provide an explicit statement of questions being addressed with reference to participants, interventions, comparisons, outcomes, and study design (PICOS).	6
**METHODS**			
Protocol and registration	5	Indicate if a review protocol exists, if and where it can be accessed (e.g., Web address), and, if available, provide registration information including registration number.	7
Eligibility criteria	6	Specify study characteristics (e.g., PICOS, length of follow-up) and report characteristics (e.g., years considered, language, publication status) used as criteria for eligibility, giving rationale.	7-8
Information sources	7	Describe all information sources (e.g., databases with dates of coverage, contact with study authors to identify additional studies) in the search and date last searched.	7-8
Search	8	Present full electronic search strategy for at least one database, including any limits used, such that it could be repeated.	7 (+ Supplemental material appendix A)
Study selection	9	State the process for selecting studies (i.e., screening, eligibility, included in systematic review, and, if applicable, included in the meta-analysis).	7-8
Data collection process	10	Describe method of data extraction from reports (e.g., piloted forms, independently, in duplicate) and any processes for obtaining and confirming data from investigators.	7-8

Data items	11	List and define all variables for which data were sought (e.g., PICOS, funding sources) and any assumptions and simplifications made.	7-8
Risk of bias in individual studies	12	Describe methods used for assessing risk of bias of individual studies (including specification of whether this was done at the study or outcome level), and how this information is to be used in any data synthesis.	8-9
Summary measures	13	State the principal summary measures (e.g., risk ratio, difference in means).	9-10
Synthesis of results	14	Describe the methods of handling data and combining results of studies, if done, including measures of consistency (e.g., I^2) for each meta-analysis.	9-10

Section/topic	#	Checklist item	Reported on page #
Risk of bias across studies	15	Specify any assessment of risk of bias that may affect the cumulative evidence (e.g., publication bias, selective reporting within studies).	10
Additional analyses	16	Describe methods of additional analyses (e.g., sensitivity or subgroup analyses, meta-regression), if done, indicating which were pre-specified.	9-10
**RESULTS**			
Study selection	17	Give numbers of studies screened, assessed for eligibility, and included in the review, with reasons for exclusions at each stage, ideally with a flow diagram.	10-11
Study characteristics	18	For each study, present characteristics for which data were extracted (e.g., study size, PICOS, follow-up period) and provide the citations.	15-16
Risk of bias within studies	19	Present data on risk of bias of each study and, if available, any outcome level assessment (see item 12).	13-16
Results of individual studies	20	For all outcomes considered (benefits or harms), present, for each study: (a) simple summary data for each intervention group (b) effect estimates and confidence intervals, ideally with a forest plot.	15-17,
Synthesis of results	21	Present results of each meta-analysis done, including confidence intervals and measures of consistency.	17-18
Risk of bias across studies	22	Present results of any assessment of risk of bias across studies (see Item 15).	17
Additional analysis	23	Give results of additional analyses, if done (e.g., sensitivity or subgroup analyses, meta-regression [see Item 16]).	17-18
**DISCUSSION**			
Summary of evidence	24	Summarize the main findings including the strength of evidence for each main outcome; consider their relevance to key groups (e.g., healthcare providers, users, and policy makers).	18-20
Limitations	25	Discuss limitations at study and outcome level (e.g., risk of bias), and at review-level (e.g., incomplete retrieval of identified research, reporting bias).	20

Conclusions	26	Provide a general interpretation of the results in the context of other evidence, and implications for future research.	20-21
**FUNDING**			
Funding	27	Describe sources of funding for the systematic review and other support (e.g., supply of data); role of funders for the systematic review.	n/a

From: Moher D, Liberati A, Tetzlaff J, Altman DG, The PRISMA Group (2009). Preferred Reporting Items for Systematic Reviews and Meta-Analyses: The PRISMA Statement. PLoS Med 6(6): e1000097. doi:10.1371/journal.pmed1000097

For more information, visit: **www.prisma-statement.org**.

Supplemental Figure 1

Funnel plot of the standardised effect size from each study (x-axis) against the standard error of effect size (y-axis)

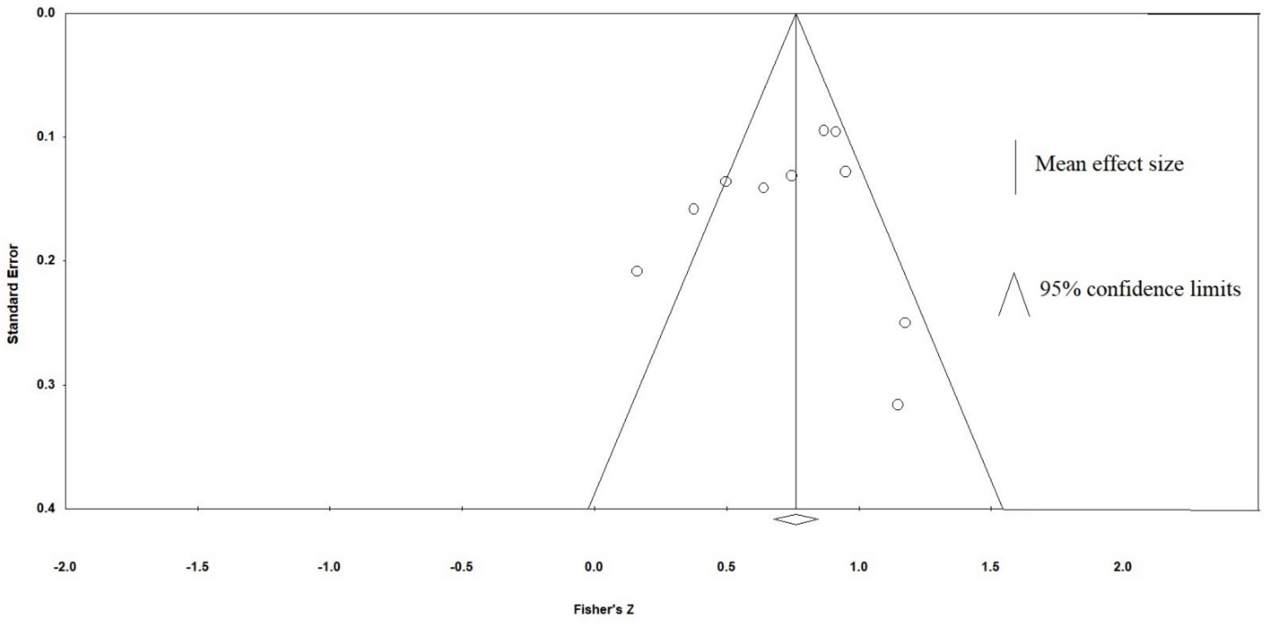

Supplement: Supplemental_material_revised_2 – Supplemental material for The relationship between intolerance of uncertainty and anxiety in autism: A systematic literature review and meta-analysis [file Supplemental_material_revised_2.pdf]
